# How COVID-19 spread varied by resident length of stay and resident–staff transmission pathways over time in US nursing homes

**DOI:** 10.1017/ash.2023.389

**Published:** 2023-09-29

**Authors:** Jessica Healy, Prabasaj Paul, Brajendra Singh, Nimalie Stone, Kara Jacobs Slifka, Rachel Slayton

## Abstract

**Background:** Pathogen transmission among staff and residents in nursing homes can vary depending on their interactions and by the amount of time a resident receives care in the facility. Understanding the relative differences in transmission rates between and among staff and residents can identify the pathways that contributed most to the spread of SARS-CoV-2 in US nursing homes. Further exploring relative differences by categorizing facilities by residents’ lengths of stay can identify priority categories for intervention. **Methods:** Using US National Healthcare Safety Network (NHSN) surveillance data on resident and staff cases, vaccination, and resident deaths during June 2020–June 2022, we estimated SARS-CoV-2 transmission among and between residents and staff. We used a Bayesian inversion of a susceptible–exposed–infected–removed–virus–death (SEIRVD) compartmental model to produce the estimates. The facilities were divided into those with median length of stay (LOS) among the residents of 10 weeks. Additional inputs included the incidence and vaccination levels of the county where each facility was located. For the compartmental model, all data were averaged to form a representative facility for each category. Transmission was estimated separately for 3 periods: (1) June 2020–March 2021 as before the SARS-CoV-2 delta variant, (2) April 2021—October 2021 during SARS-CoV-2 delta variant dominance, and (3) November 2021—June 2022 during the prevalence of the SARS-CoV-2 omicron variant. **Results:** Regardless of facility category, transmission was highest from staff to residents or resident to resident (Fig.). These estimates of transmission were highest during the pre–SARS-CoV-2 delta variant phase. Transmission in that phase was highest in the facilities with LOS >10 weeks from staff to residents at 0.88 per week (95% credible interval [CrI], 0.06–1.85), in the facilities with LOS 6–10 weeks from staff to residents at 0.68 per week (95% CrI, 0.03–1.78), and in the facilities with LOS <6 weeks between residents at 0.47 per week (95% CrI, 0.02–0.95). **Conclusions:** Staff-to-resident or resident-to-resident transmission were the dominant pathways of spread of SARS-CoV-2 across the periods or the facility categories. Facilities with LOS 6 weeks or longer had higher median transmission estimates across the periods and transmission routes compared to facilities with LOS less than 6 weeks, implying that when prioritization of intervention resources is needed, facilities caring for populations with longer stays could be prioritized.

**Figure:**
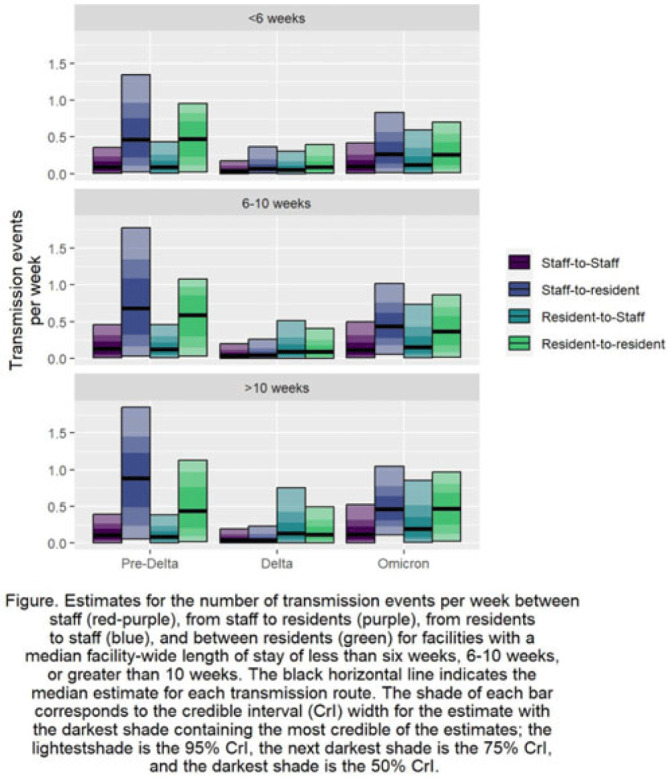

**Disclosures:** None

